# Tissue-Specific Transcript Profiling for ABC Transporters in the Sequestering Larvae of the Phytophagous Leaf Beetle *Chrysomela populi*

**DOI:** 10.1371/journal.pone.0098637

**Published:** 2014-06-02

**Authors:** Anja S. Strauss, Ding Wang, Magdalena Stock, René R. Gretscher, Marco Groth, Wilhelm Boland, Antje Burse

**Affiliations:** 1 Max Planck Institute for Chemical Ecology, Beutenberg Campus, Hans-Knoell-Str. 8, D-07745 Jena, Thuringia, Germany; 2 Leibniz Institute for Age Research – Fritz Lipmann Institute, Beutenbergstr. 11, D-07745 Jena, Thuringia, Germany; Hungarian Academy of Sciences, Hungary

## Abstract

**Background:**

Insects evolved ingenious adaptations to use extraordinary food sources. Particularly, the diet of herbivores enriched with noxious plant secondary metabolites requires detoxification mechanisms. Sequestration, which involves the uptake, transfer, and concentration of occasionally modified phytochemicals into specialized tissues or hemolymph, is one of the most successful detoxification strategies found in most insect orders. Due to the ability of ATP-binding cassette (ABC) carriers to transport a wide range of molecules including phytochemicals and xenobiotics, it is highly likely that they play a role in this sequestration process. To shed light on the role of ABC proteins in sequestration, we describe an inventory of putative ABC transporters in various tissues in the sequestering juvenile poplar leaf beetle, *Chrysomela populi*.

**Results:**

In the transcriptome of *C. populi*, we predicted 65 ABC transporters. To link the proteins with a possible function, we performed comparative phylogenetic analyses with ABC transporters of other insects and of humans. While tissue-specific profiling of each ABC transporter subfamily suggests that ABCB, C and G influence the plant metabolite absorption in the gut, ABCC with 14 members is the preferred subfamily responsible for the excretion of these metabolites *via* Malpighian tubules. Moreover, salicin, which is sequestered from poplar plants, is translocated into the defensive glands for further deterrent production. In these glands and among all identified ABC transporters, an exceptionally high transcript level was observed only for *Cpabc35* (*Cpmrp*). RNAi revealed the deficiency of other ABC pumps to compensate the function of *Cp*ABC35, demonstrating its key role during sequestration.

**Conclusion:**

We provide the first comprehensive phylogenetic study of the ABC family in a phytophagous beetle species. RNA-seq data from different larval tissues propose the importance of ABC pumps to achieve a homeostasis of plant-derived compounds and offer a basis for future analyses of their physiological function in sequestration processes.

## Introduction

Lipid bilayers form efficient barriers for cellular partitioning. The translocation across these membranous barriers is crucial for many aspects of cell physiology, including the uptake of nutrients, the elimination of waste products, or energy generation and cell signaling. The ATP-binding cassette (ABC) transporters constitute one of the largest families of membrane translocators [1]. The core functional unit of ABC proteins consists of four domains: two cytoplasmic domains containing the highly conserved nucleotide-binding domains (NBDs), which are responsible for the ATP hydrolysis needed to provide energy for the transport cycle, and two transmembrane domains (TMDs), each in most cases composed of six membrane-spanning helices, which impart substrate specificity and translocation [2]–[4]. The NBDs harbor several conserved sequence motifs from N- to C-terminus. These are the Walker A motif (also called P-loop) which is glycine-rich, a flexible loop with a conserved glutamine residue (Q-loop), the ABC signature (LSGGQ) motif (also called C-loop), the Walker B motif, and a conserved histidine residue (His-switch). The ABC signature motif is diagnostic for this family as it is present only in ABC transporters, while Walker A and B motifs are found in many other ATP-utilizing proteins. The domains are encoded by separate genes, either by genes encoding one NBD and one TMD whose products dimerize to form the functional transporter, or by genes encoding two NBDs and two TMDs on a single polypeptide.

In eukaryotic genomes, ABC genes are widely dispersed and highly conserved between species, indicating that most of these genes have existed since the beginning of eukaryotic evolution [5]–[8]. ABC transporters can be classified into subfamilies according to sequence homology and domain topology. The existing eukaryotic genes have been grouped into major subfamilies, termed from ABCA to ABCI [1], [9]. Both subfamilies H and I are not present in humans. The subfamily ABCH was defined after the analysis of the genome of the fruit fly *Drosophila melanogaster* and was found in other invertebrates and zebrafish to date. The subfamily ABCI is limited to plants [10]. Most ABC proteins transport a wide range of compounds, either within the cell as part of a metabolic process into an intracellular compartment (e.g. endoplasmic reticulum (ER), mitochondria, and peroxisomes) or outside the cell for transport processes to other organs. In humans, the known functions of ABC transporters include cholesterol and lipid transport, multidrug resistance, antigen presentation, mitochondrial iron homeostasis and the ATP-dependent regulation of ion channels [11]–[14]. Owing to the importance of ABC transporters for cell functions, they are still extensively investigated in many eukaryotes. In insects, one of the best studied ABC proteins is White, which is crucial for pigment transfer in insect eyes [15]–[20]. As is known for *D. melanogaster*, ABC transporters facilitate translocation of attractants for germ cell migration [21] or participate in the modulation of the molting hormones’ (ecdysteroids’) signaling in insect tissues [22]. Furthermore, they seem to be frequently implicated in insecticide resistance [23], [24], such as in the DTT tolerance of the *Anopheles* mosquitoes which transmit malaria agents [25] or in the tolerance against pest control toxins from *Bacillus thuringiensis* which is reported of lepidopterans [26], [27].

Although ABC transporters were previously analyzed in several insect species at genome-wide level [28], [29], profiles of the transcript levels of ABC transporters in non-model insects are not available to date. For this study we analyzed the transcriptomic data with regard to ABC transporters in a phytophagous leaf beetle species. Leaf beetles (Chrysomelidae *sensu lato*; including the seed beetles Bruchidae) constitute together with the Cerambycidae (longhorn beetles) and the Curculionoidea (weevils) the largest beetle radiation. These are known as “Phytophaga” and represent roughly 40% of all the 350,000 described species [30]. Leaf beetles mainly feed on green plant parts. The species of the leaf beetle taxon Chrysomelina, for example, are adapted to use host plants’ leaves as a food source during their whole life cycle [31]. Therefore, they have to be protected against both, the noxious effect of plant secondary metabolites and attacks by their enemies. Some species evolved the ability to exploit the phytochemicals for their own chemical defense [32]–[34]. The larvae of the poplar leaf beetle *Chrysomela populi*, for example, take up the phenolglucoside salicin from salicaceaous food plants. This precursor salicin is transported into nine pairs of exocrine, dorsal glands [35], [36], where the compound is converted into salicylaldehyde – a potent, volatile deterrent that repels predators and prevents fatal microbial infections [34], [37], [38]. This process of sequestration involves a complex influx-efflux transport network which guides plant-derived glucosides through the insect body [39].

Although sequestration is a widespread phenomenon attributed to many insect orders, we recently identified the first example of a transport protein essential for the translocation of phytochemicals in insects [40]. The transporter belongs to the ATP-binding cassette transporter family and functions in the defensive exocrine glands of juvenile poplar leaf beetles. Thus, the comprehensive analysis of putative ABC transporters in the phytophagous *C. populi* larvae provides implications for further studies on the predicted physiological functions of this transporter class in sequestering insects, such as the incorporation and excretion mechanisms of toxic compounds. For this reason, we present a complete inventory of ABC transporters based on available *C. populi* transcriptome sequences. Detailed sequence comparisons of members of each subfamily with those of humans, the red flour beetle *Tribolium castaneum* and other insects reveal their correspondences. We, additionally, studied the expression profiles of ABC encoding transcripts in various tissues by using next-generation sequencing in juvenile *C. populi* and propose a function of ABC pumps in the sequestration process.

## Materials and Methods

No specific permissions were required for the locations/activities. *Chrysomela populi* is listed neither as endangered nor as protected species in Germany. Manuela Baerwolff from the Thueringer Landesanstalt für Landwirtschaft, Referat 430 Nachwachsende Rohstoffe, D-07778 Dornburg-Camburg, Apoldaer Str. 4 allowed us to collect the beetles in the area mentioned in the manuscript. GPS coordinates are stated in the method section.

### Rearing and Maintaining of *C. populi*

*C. populi* (L.) was collected near Dornburg, Germany (+51°00′52.00″, +11°38′17.00″) on *Populus maximowiczii* × *Populus nigra*. The beetles were kept in a light/dark cycle of 16 h light and 8 h darkness (LD 16/8) at 18°C±2°C in light and 13°C±2°C in darkness.

### RNA Isolation, Library Construction and Sequencing

Tissue samples from five *C. populi* larvae per biological replicate were collected as described by Bodemann *et al.* [41]. Total RNA was extracted from defensive glands, fat body, Malpighian tubules and gut tissue with the RNAqueous Micro Kit (Ambion, Life Technologies, Carlsbad, California, USA) according to the manufacturers’ instructions with the exception of 1% (v/v) ExpressArt NucleoGuard (Amplification Technologies, Hamburg, Germany) added to the lysis buffer. The RNA integrity was validated by electrophoresis on RNA 6000 Nano labchips on a Bioanalyzer 2100 (Agilent Technologies, Santa Clara, California, USA). RNA concentrations were determined by employing a NanoView (GE-Healthcare, Chalfont St Giles, UK). Up to 5 µg of total RNA was then used for library preparation using TruSeq RNA Sample Prep Kit (Illumina, San Diego, California, USA) according to the manufacturer’s description. RNA sequencing (RNA-seq) for three biological samples per prepared tissue was done using next-generation sequencing technique [42] on a HiSeq2000 (Illumina, San Diego, California, USA) in 50-bp single read mode (two or three samples multiplexed in one lane).

Pooled total RNAs from adults (two males, two females), one pupa, and nine first- to third-instar larvae were used for paired-end sequencing. Up to 5 µg of total RNA was then used for library preparation using TruSeq RNA Sample Prep Kit (Illumina Inc., San Diego, USA) according to the manufacturer’s description. Afterwards, the fragmentation step during library preparation of these was set to four minutes. This library was sequenced using a GAIIx (Illumina Inc., San Diego, USA) in 150-bp paired-end mode in one sample per lane. All reads were extracted in FastQ format and used for further analysis.

### *De novo* Assembly of *C. populi*’s Transcriptome

To obtain the transcript catalogue of *C. populi*, the paired-end reads were *de novo* assembled by applying the open source tool Trinity v2012–03–17 [43] with the following parameters: minimal contig length of 300 bp and the paired fragment length limited to 500 bp. In order to reconstruct full-length transcripts, we used the software TGICl (vJan.2009) [44] to reassemble the transcriptome output from Trinity with a minimum overlap length of 100 bp and sequence similarity of 90 percent. A summary of these results is given in Table S1. The raw sequence data are stored in the Sequence Read Archive (SRA) of the National Center for Biotechnology Information (NCBI) with the accession number SRA106166. The corresponding BioProject is PRJNA212154.

### Annotation of *De* *novo* Assembled Transcript Library and Identification of ABC Transporters

We annotated the above mentioned transcript catalogue by translating the cDNAs of the putative transcripts into all six possible open reading frames. This was achieved by applying transeq which is part of the EMBOSS package (v6.3.1). Afterwards, the protein sequences were searched against the Pfam database (update, Jan 2013) with an e-value cut-off of 1e-5 [45], [46]. 102 hits were obtained that belong to the protein family “PF00005” (ABC_tran domain). Next, we identified 12 sequences highly similar to obligate intracellular Microsporidia parasites found by BLASTx against the non-redundant protein sequence database (at NCBI). The database search revealed, for example, that the sequence named Msp1 displays 69% identity to the protein of *Nosema ceranae* (XP_002996720.1). Other tested sequences which exhibited similarities between 48% and 93% identity to members of the genus *Nosema* and other Microsporidia were considered as sequences derived from these intracellular parasites. Because the presumed parasite in *C. populi* has not yet been taxonomically classified, we specified these sequences which we have included into our phylogenetic study as Msp1–5 (for Microsporidia). In general, Microsporidia are widespread parasites also reported from Chrysomelidae [47], [48], including from *Chrysomela scripta* (a close relative of *C. populi*) whose tissues were infected with *Nosema scripta* [49]. In the future, the probable infection of *C. populi* with Microsporidia and their effects on the host need to be clarified.

For the identification of NBDs in 90 ABC transporters (after the removal of the presumed mircosporidian sequences), firstly, the highly conserved NBDs of the human (ABCA-ABCG, 48 amino acid sequences of NBD) and fruit fly (ABCH, 3 amino acid sequences of NBD) ABC transporters were retrieved from GenBank (NCBI) and chosen as ‘homology search targets’. Then, the long coding sequence for each annotated beetle ABC transporter was determined by using getorf of the EMBOSS tools. Afterwards, these longest coding sequences and the chosen ‘homology search targets’ were aligned by applying the multiple sequence alignment program MAFFT v7.01 [50] (using option E-INS-i). Transcripts containing all five motifs of NBDs with roughly 170 amino acids were kept. Secondly, the remaining ABC transporter transcripts with incomplete motifs were checked again. Their six possible protein sequences were aligned to the chosen ‘homology search targets’ (with the same parameter E-INS-i, MAFFT). All sequences containing at least four motifs of NBDs and having a sequence length of more than 130 amino acids were selected and added to the other sequences for further studies. These incomplete sequences might be due to the stringent settings while assembling them. For our assembly we have tested different parameter settings including lower stringency. However, we have realized that this let to contiguous sequences consisting of several transcripts that have been assembled together although they do not belong to each other. Therefore, we have decided to choose the stringent parameters which ensure obtaining unique rather than complete sequences. We have aligned our incomplete sequences with the most identical once from *T. castaneaum* and observed that the sequences from *T. castanaeum* encode complete proteins. Based on this, we assumed that our sequences might also encode complete proteins, and that only due to limitations in the *de novo* assembly we did not obtain complete coding regions. Therefore, we did not exclude these sequences from our analyses. After removal of isoforms, the resulting beetle sequences were deposited as Transcriptome Shotgun Assembly project at DDBJ/EMBL/GenBank under the accession GARF00000000. The version described in this paper is the first version, GARF01000000.

### Calculation of Phylogenetic Trees

The protein sequences were aligned by the G-INS-i methods from MAFFT with default parameters. To calculate the phylogenetic tree RAxML v7.2.8 [51], a program based on maximum-likelihood inference, was used. In RAxML, the best fit model of protein evolution was RTREVF with gamma distribution for modeling rate heterogeneity. The best fit model was determined by the best likelihood score under GAMMA (perl script ProteinModelSelection.pl, which was downloaded from http://sco.h-its.org/exelixis/hands-On.html). The maximum-likelihood phylogenetic tree was reconstructed with a bootstrap test of 1000 replicates in RAxML.

For phylogenetic analysis of the ABC transporter subfamilies, we used the same methods along with sequences of *T. castaneum*, the most closely related model species to *C. populi*. The ABC transporter protein sequences of *T. castaneum* were retrieved from Broehan *et al.* [52] with the identical designations. Further, we included homologous sequences from human, *Bombyx mori*, *D. melanogaster*, *Apis mellifera*, *Culex quinquefasciatus*, *Dendroctonus ponderosae*, and from a Microsporidia species into our calculations. If not stated in the phylogenetic trees, the accession numbers of these sequences are listed in Table S2.

### Expression Profiling of Putative ABC Transporter Transcripts

Each 50-bp single-read dataset of four tissues (gut, defensive glands, fat body, Malpighian tubules) contained three biological replicate samples. The raw sequence data are stored in the SRA of the NCBI with the accession numbers listed in Table S3. The corresponding BioProject is PRJNA212154.

To compare the transcript expression levels of the four tissues, we mapped the RNA-seq reads onto the (*de novo* assembled) transcriptome of *C. populi* with the open source tool Bowtie v0.12.7 [53] using default parameters. Afterwards, the R package DESeq [54], [55] (which is part of the Bioconductor package [56]) was used to detect differentially expressed transcripts in the four different tissues.

Based on the Lander/Waterman equation [57], the average coverage per base in each transcript of each biological replicate was separately computed. The mean values of average coverage of each replicate for each tissue, respectively, were compared to show the expression levels of tissues (see Table S4 for normalized data). To compare these results with quantitative real-time PCR measurements, we normalized the output from DESeq to the standards *CpeIF4a* and *CpEF1alpha* (see Table S5 for the accession numbers of normalization genes), which were used in quantitative real-time PCR, as described by Livak and Schmittgen [58].

### Quantitative Real-time PCR (qPCR)

Total RNA was extracted from larval tissue using an RNeasy MINI kit (Qiagen, Hilden, Germany). Complementary DNA was synthesized from DNA-digested RNA using SuperScript III reverse transcriptase (Invitrogen, Life Technologies, Carlsbad, California, USA). Real-time PCR was performed using Brilliant II SYBR Green qPCR Master Mix (Agilent Technologies, Santa Clara, California, USA) according to the manufacturer’s instructions and the Mx3000P Real-Time PCR system (Agilent Technologies, Santa Clara, California, USA). *CpeIF4a* and *CpEF1alpha* expression were used to normalize transcript quantities (see Table S5 for primer sequences). Running conditions: 3′ 94°C, 40 cycles (30″ 94°C; 30″ 60°C), melting curve with 1°C increase 60–95°C. Analyses were performed according to the MIQE-guidelines [59].

### RNA Interference of *Cpabc35* (*CpMRP*) in *C. populi* Larvae

The most abundant ABC transporter derived from the glandular tissue (*Cpabc35* (*Cpmrp*)) [40] was analyzed *via* RNAi experiments. The sequence-verified plasmid pIB-*Cp*MRP was used to amplify a 730-bp fragment of *Cpabc35* dsRNA. As control, a *gfp* sequence was amplified from pcDNA3.1/CT-GFP-TOPO (Invitrogen, Life Technologies, Carlsbad, California, USA). The amplicons were subjected to *in vitro* transcription assays according to the instructions of the MEGAscript RNAi kit (Ambion, Life Technologies, Carlsbad, California, USA; see Table S5 for primer sequences). The resulting dsRNA was eluted three times with 50 µl of injection buffer (3.5 mM Tris-HCl, 1 mM NaCl, 50 nM Na_2_HPO_4_, 20 nM KH_2_PO_4_, 3 mM KCl, 0.3 mM EDTA, pH 7.0) after nuclease digestion. The quality of dsRNA was checked by TBE-agarose-electrophoresis.

First-instars of *C. populi* (3–4 days after hatching) with 3–5 mm body length (chilled on ice) were injected with 0.25 µg of dsRNA by using a nanoliter microinjection system (Nanoliter 2000 Injector, World Precision Instruments, Sarasota, Florida, USA). Injections were made into the hemolymph next to the ventral side between the pro- and mesothorax. Differential expression in the glandular tissue was analyzed 10 days after RNAi treatment by using RNA-seq. Two biological replicates (pool of glandular tissue of 3 larvae, each) compared to two biological replicates of *gfp*-control samples [42] were sequenced on a HiSeq2500 (Illumina, San Diego, California USA) in 50-bp single read mode (two or three samples multiplexed in one lane). The raw sequence data are stored in the SRA of the NCBI with the accession numbers listed in Table S3. The corresponding BioProject is PRJNA212154. All short reads again were extracted in FastQ format for further analysis.

### Analysis of Differentially Expressed Genes in the Glandular Tissue of Rnai-silenced *C. populi* Larvae

The short reads (sequenced in 50 bp single-mode) from the glandular tissue of the RNAi-silenced (2 samples) as well as ds*gfp*-injected (2 samples) *C. populi* larvae were mapped onto *C. populi*'s transcriptome using Bowtie [53]. The mapping results for the ABC transporter transcripts were subjected to DESeq statistical analysis [54], [55] by reading them into R statistics software. Transcript counts were normalized to the effective library size. Afterwards, the negative binomial testing was carried out to identify differentially expressed genes (DEGs). All those genes were determined as differentially expressed when having an adjusted p-value less than 0.1. From all DEGs, the annotated ABC transporters were selected and checked for co-regulation.

## Results and Discussion

### Identification of Putative ABC Transporters Encoded in the Transcript Catalogue of *C. populi*

In our study, we focused on the distribution of ABC transporters in the different tissues of juvenile *C*. *populi* to assign a function to each transcript related to a certain tissue. For this purpose, we first identified potential ABC transporters in the *de novo* assembled transcript catalogue of the poplar leaf beetle. The transcriptome sequences were translated into all possible amino acid sequences and further processed as described in the method section. As a result, we predicted 65 ABC transporters for *C. populi*. This corresponds with previous studies on insects reporting, for example, 73 ABC transporter genes in the genome of *T. castaneum* [52], 44 in *Anopheles gambiae* [29], 56 in *D. melanogaster*, 43 in *A. mellifera*, or 51 in *B. mori* [28]. The *C. populi* sequences were given temporary designations as numbered series in the form of *Cp*ABCxx (Table S4).

### Phylogenetic Analysis of the Putative ABC Transporters

**Figure 1:**
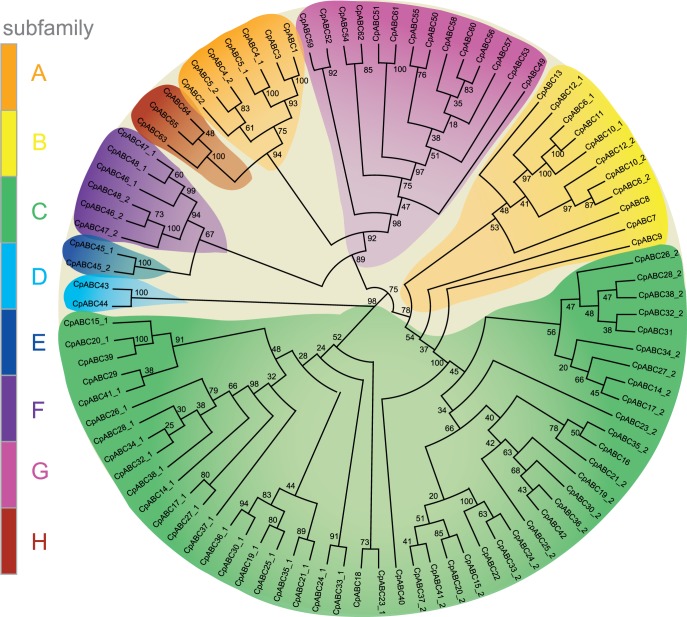
Eight subfamilies of 65 putative ABC transporters of *C. populi*. Some transporters contain two NBDs (NBD1 as *Cp*ABCX_1 and NBD2 as *Cp*ABCX_2), others contain only one NBD. Numbers at nodes represent bootstrap values.

Based on structural and functional similarity, ABC transporters in general can be grouped into subfamilies. In order to predict the subfamilies for the 65 identified ABC proteins in *C. populi*, we used their extracted NBDs for the multiple sequence alignments and then calculated the phylogenetic tree. Similarly to other insects and eukaryotes, we were able to show a division of the predicted transporters into 8 subfamilies (A–H) (Figure 1; Table 1). Members of ABCA, ABCE/F, ABCG and ABCH form distinct branches (bootstrap value ≥75 percent). ABCH forms a sister group of ABCA. The ABCC family segregates into two groups: ABCC1 contains NBDs1 and shows a similarity to the ABCD subfamily; ABCC2 contains NBDs2 and shows a similarity to the ABCB subfamily. Among the 65 putative ABC transporters from *C. populi* we identified full, half and incomplete transporters. The distribution of domains in the sequences is shown in detail for each subfamily in Table 2 and for each sequence in Table S6.

**Table 1:** Subfamilies of ABC transporters in eight eukaryotic species (Numbers were derived from [115]).

Species	ABCA	ABCB	ABCC	ABCD	ABCE	ABCF	ABCG	ABCH	total
*S. cerevisiae*	0	4	6	2	2	6	10	0	30
*C. elegans*	7	24	9	5	1	3	9	0	58
*D. pulex*	4	7	7	3	1	4	24	15	65
*T. urticae*	10	4	39	2	1	3	23	22	103
*D. melanogaster*	10	8	14	2	1	3	15	3	56
*T. castaneum*	10	6	35	2	1	3	13	3	73
*C. populi*	5	8	29	2	1	3	14	3	65
*H. sapiens*	12	11	12	4	1	3	5	0	48

**Table 2:** Distribution of domains in the eight ABC transporter subfamilies of *C. populi*.

	Full-trans	Half-trans	2*NBD	2*NBD+1*TMD	1*NBD	1*NBD+2*TMD	total
**ABCA**	2	1	0	0	2	0	**5**
**ABCB**	3	5	0	0	0	0	**8**
**ABCC**	18	4	0	3	3	1	**29**
**ABCD**	0	2	0	0	0	0	**2**
**ABCE**	0	0	1	0	0	0	**1**
**ABCF**	0	0	3	0	0	0	**3**
**ABCG**	0	12	0	0	2	0	**14**
**ABCH**	0	3	0	0	0	0	**3**
**total**	23	27	4	3	7	1	**65**

Full-trans, full transporters; Half-trans, half transporters; NBD, nucleotide-binding domain; TMD, transmembrane domain; 2*NBD+1*TMD, two NBDs and one TMD (example).

Next, we integrated human and other insect sequences into our phylogenetic trees. This allowed us to group the putative *C. populi* ABC transporters with functionally characterized proteins and, thus, to propose a substrate for the beetles’ proteins.

**Figure 2:**
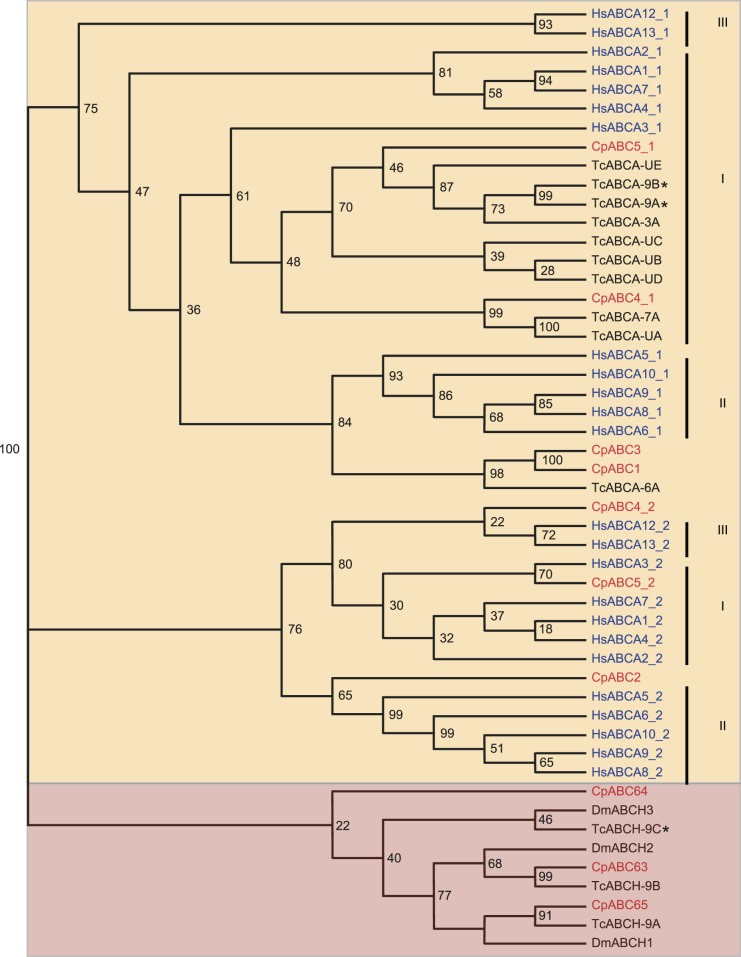
Phylogenetic tree of subfamilies ABCA (orange) and ABCH (pink). Some transporters contain two NBDs (NBD1 as *Cp*ABCX_1 and NBD2 as *Cp*ABCX_2), others contain only one NBD. Red, *C. populi* (Cp); blue, *H. sapiens* (Hs); black, *T. castaneum* (Tc), *D. melanogaster* (Dm). *, *T. castanaeum* with phenotype after RNAi. Numbers at nodes represent bootstrap values.

In the case of **subfamily A**, its members in humans are full transporters and implicated in the transport processes of phospholipids, sterols, sphingolipids, bile salts, retinal derivatives (restricted to ABCA4) and other lipid conjugates indispensable for many biological processes [11], [60]–[63]. In insects, both full and half transporters were identified whose physiological function, however, is not yet understood [28]. In *C. populi* we predicted five transporters. According to our phylogenetic analysis, ABCA proteins segregate into one branch containing NBD1 and one branch with NBD2 (Figure 2). Human ABCAs form three groups (I, ABCA1-4, 7; II, ABCA5, 6, 8-10; III, ABCA12 and 13) which are particularly distinguishable in the NBD2 branch with bootstrap values ≥76 percent. Considering the beetles’ homologs, the tree shows that the majority of *C. populi* and *T. castaneum* sequences seem to cluster to human ABCA3 which results in an expansion of group I.

The **ABCB** subfamily contains ABCB1 (MDR1/P-glycoprotein) which is the first characterized human ABC transporter to confer multidrug resistance (MDR) in cancer cells [64]–[66] and which has been intensively studied ever since the discovery of cross-resistances after selection with chemotherapeutics [67]–[71]. Later studies revealed additional ABCB transporters as MDR proteins. Besides xenobiotic extrusion (ABCB1, 5, 8) [72]–[74], ABCB members are also known in human biology for the translocation, for example, of phosphatidylcholine (ABCB4) [75], bile acids (ABCB11) [76], peptides (TAP1:TAP2 (antigen processing in the adaptive immune system), TAPL, mitochondrial ABCB10) [77], metabolites of the heme synthetic pathway (ABCB6) [78], or iron (mitochondrial ABCB7 and 8) [79]–[82]. In insects, several examples suggest the involvement of P-glycoproteins in the resistance to insecticides used for crop protection [23], [24], [83]–[91]. However, only few P-glycoprotein-like genes have been linked to a xenobiotic substrate such as *Mdr49* and *Mdr65* of *D. melanogaster* with tolerance against colchicine and α-amanitin, respectively [92], [93]. Mdr49 can act also as transporter for a germ cell attractant in fruit flies [21]. Recent studies on lepidopteran species revealed that a P-glycoprotein-like transporter mediates the efflux of cardenolides in the nerve cord and thereby prevents the interactions of these toxins with the susceptible target site of Na+/K+-ATPases [94].

**Figure 3:**
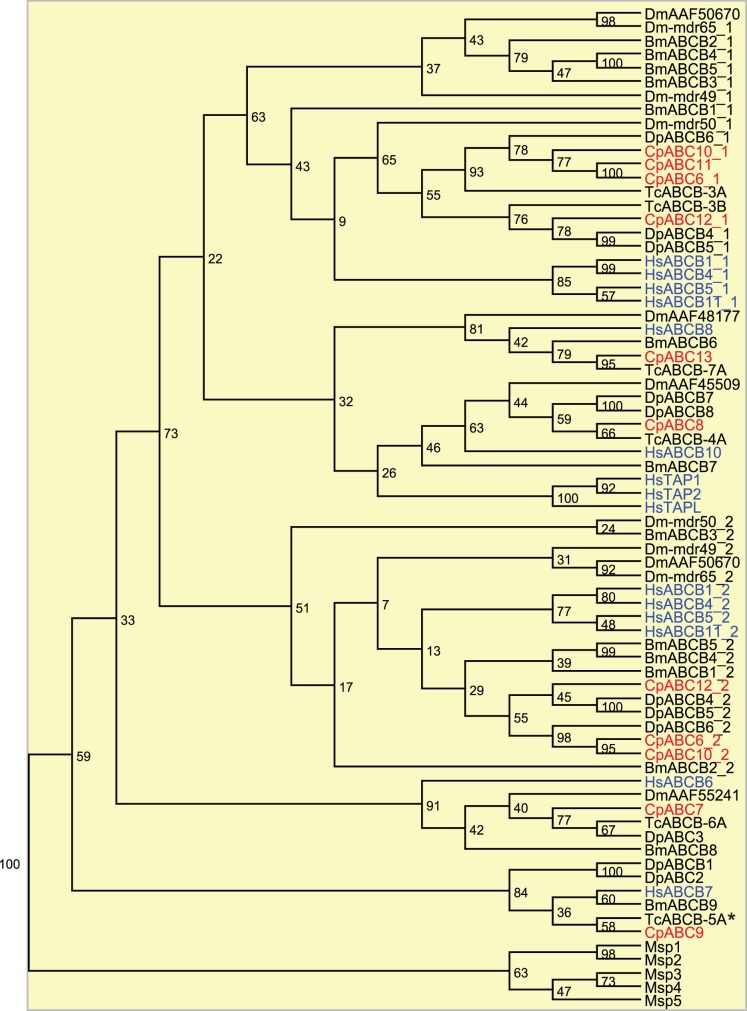
Phylogenetic tree of subfamily ABCB. Some proteins contain two NBDs (NBD1 as *Cp*ABCX_1 and NBD2 as *Cp*ABCX_2), others contain only one NBD. Red, *C. populi* (Cp); blue, *H. sapiens* (Hs); black, *T. castaneum* (Tc), *D. ponderosae* (Dp), *D. melanogaster* (Dm), *B. mori* (Bm); Microsporida (Msp). *, *T. castanaeum* with phenotype after RNAi. Numbers at nodes represent bootstrap values.

Similar to other insects, the eight sequences from *C. populi* encode full and half transporters. Bootstrapping of the ABCB phylogenetic tree in Figure 3 and Figure S1 (together with ABCC) produced nodes weakly supporting segregation of this subfamily containing human and insect ABCB sequences. Based on our phylogenetic analysis, we found no homologs to TAP sequences (bootstrap value of 100 percent) in the insects, but insect homologs to the other human peptide transporters were identified. In accordance with the literature, we can also speculate that TAPL is the ancestor of the TAP family [95].

**Figure 4:**
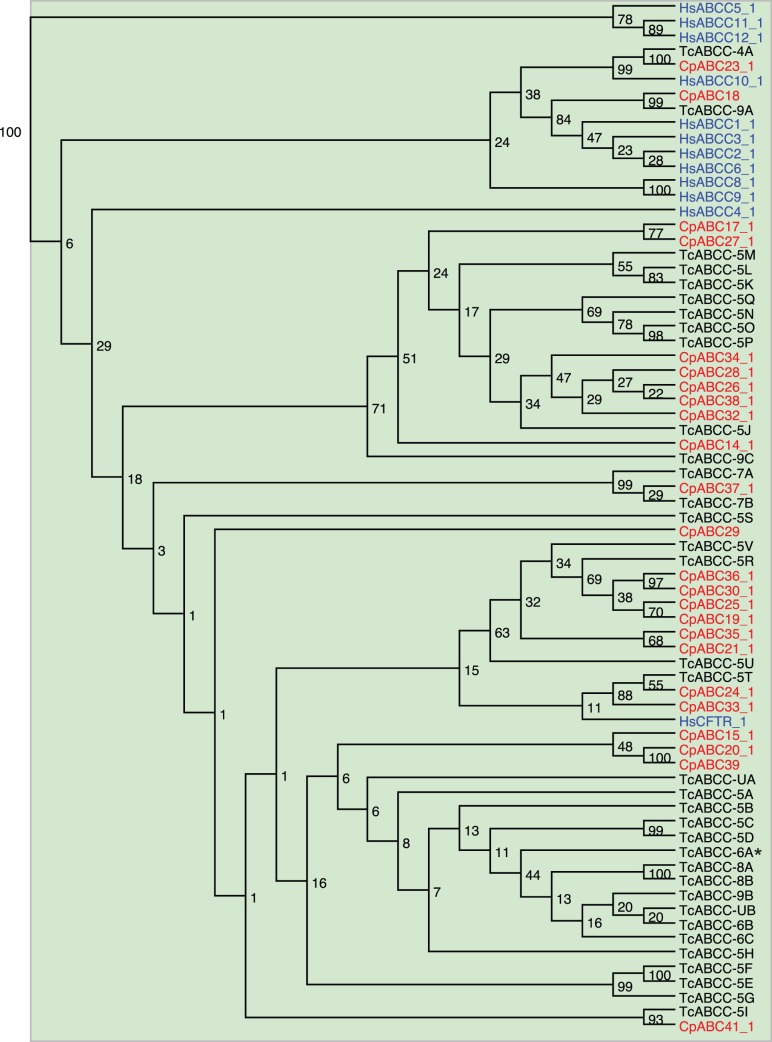
Phylogenetic tree of NBDs1 from subfamily ABCC. Red, *C. populi* (Cp); blue, *H. sapiens* (Hs); black, *T. castaneum* (Tc). *Hs*ABC8 and 9 are sulfonylurea receptors (SUR); *, *T. castanaeum* with phenotype after RNAi. Numbers at nodes represent bootstrap values.

Full transporters of striking functional diversity are found in the **ABCC** subfamily. In humans thirteen ABCC members were identified, nine of which are referred to as multidrug resistance proteins (MRP) (‘short’ MRPs (ABCC4, −5, −11 and −12); ‘long’ MRPs (ABCC1, −2, −3, −6 and −10) [13], [96], [97]. Some ABCC members not considered as MRPs have unique functions. The cystic fibrosis transmembrane conductance regulator (CFTR/ABCC7), for example, functions as an epithelial ATP-gated chloride channel [98], [99]. ABCC8 and ABCC9 are assembled as sulfonylurea receptors (SUR) into ATP-sensitive K^+^ channels and are coupled to the gating mechanism of the ion-conducting pore [100]. In insects, ABCC members are thought to be involved in the translocation of xenobiotics and phytochemicals [23]–[26], [40], [91], [101]. As observed in the red flour beetle *T. castaneum* and the spider mite *Tetranychus urticae*, the ABCC subfamily in *C. populi* with 29 putative members has undergone an expansion (Table 1). In our phylogenetic tree, the NBDs1 and NBDs2 form distinct branches (bootstrap value of 100 percent; Figure S1). The human ‘short’ MRPs ABCC5, 11, and 12 are clearly separated from all other tested sequences (Figure 4). The vast majority of insect sequences cluster together with human CFTR, SURs and multidrug resistant proteins, such as ‘long’ MRPs and ABCC4 implying a broad substrate spectrum of these proteins (Figure 4 and S1). Into this group falls also *Cp*ABC35 (*Cp*MRP) which is known to translocate phytochemicals including salicin. A substrate for any other insect homolog in this group has not been determined to date.

Members of the **ABCD** subfamily are involved in the translocation of fatty acids into peroxisomes [102]. The ABC transporters are half-size and assemble mostly as a homodimer after posttranslational transport to peroxisomal membranes. ABCD4 is not a peroxisomal membrane protein but an ER-resident protein that mediates translocation of lipid molecules essential for lipid metabolism in the ER [103]. Unlike humans but like all other sequenced insects *C. populi* contains two ABCD half transporters. Because they are homologous to the human peroxisomal and *T. castaneum* transporters, a similar function can be inferred in poplar leaf beetles. No insect sequence could be grouped to ABCD4 (Figure S2).

The **ABCE** and **ABCF** proteins comprise a pair of linked NBDs but lack TMDs. Therefore, they are not involved in molecule transport, but they are active in a wide range of other functions pivotal for cell viability. For example, the human ABCE1 not only acts as a ribonuclease L inhibitor, it also regulates RNA stability, viral infection, tumor cell proliferation, anti-apoptosis, translation initiation, elongation, termination, and ribosome recycling [104]. In *D. melanogaster*, the ABCE homolog Pixie plays a catalytic role in the assembly of protein complexes required for translation initiation [105]. All genomes of multicellular eukaryotes analyzed to date possess one ABCE gene [106]. In the transcript catalogue of *C. populi*, one complete ABCE protein has been predicted. The NBDs of *Cp*ABC45 are highly conserved with the respective NBDs of the human ABCE1 and *T. castaneum Tc*ABCE-3A (Figure S2). Among the subfamily ABCF involved in translation initiation and elongation in humans [106], we found in *C. populi* three putative members each with two NBDs that are highly similar to the transporters of human and *T. castaneum* suggesting functional proteins used in similar physiological processes in the cell.

The **ABCG** subfamily in humans is comprised of five half transporters. While the homodimer ABCG2 is a multidrug transporter with a wide substrate specificity [72], the homodimers ABCG1 and ABCG4 and the heterodimer ABCG5:ABCG8 translocate cholesterol and other sterole derivatives [107]–[110]. In insects, ABCG transporters are essential for the translocation of ommochromes for the pigmentation of eyes and body coloration. In *D. melanogaster*, for example, the half transporter White forms heterodimers with Scarlet or Brown, each of which is responsible for the transport of another type of ommochrome precursor to pigment granules [15]–[17]. In silkworms, White-orthologs (Bm-ok) are responsible for the translocation of uric acid for accumulation in urate granules in epidermal cells, resulting in opaque white coloration of the larval skin [20], [111]. In *D. melanogaster*, E23 encodes a transporter capable of modulating the ecdysone response with consequences for the circadian transcription of clock genes [22], [112].

**Figure 5:**
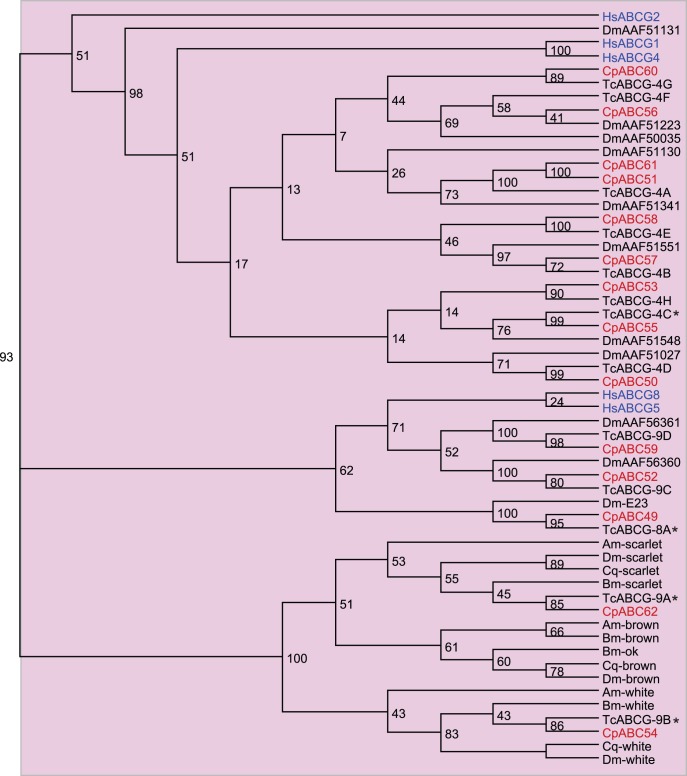
Phylogenetic tree of subfamily ABCG. Red, *C. populi* (Cp); blue, *H. sapiens* (Hs); black, *T. castaneum* (Tc), *D. melanogaster* (Dm), *B. mori* (Bm), *A. mellifera* (Ap), *C. quinquefasciatus* (Cq). *, *T. castanaeum* with phenotype after RNAi. Numbers at nodes represent bootstrap values.

The phylogenetic analysis revealed that the majority of the chosen insect sequences, including predicted ABCG proteins from *C. populi*, cluster together with the human ABCG1 and ABCG4 (bootstrap value of 98 percent) (Figure 5). Several insect ABCG candidates form a branch with the human ABCG5:ABCG8. Also E23 from *D. melanogaster* clusters in this branch together with *Tc*ABCG-8A. Silencing of *Tc*ABCG-8A resulted in molting defects, premature compound eye development, aberrant wing development and lethality, suggesting a function in the regulation of ecdysteroid-mediated effects [52]. Because *Cp*ABC49 is homologous to *Tc*ABCG-8A and *Dm*-E23, it allows the expectation of a similar function for this protein in *C. populi*. In addition, the insect ABCG proteins (White, Brown, and Scarlet) involved in the transfer of ommochrome precursors form a separate branch (bootstrap value of 93 percent). In accordance with the observation of *T. castaneum* [52], in *C. populi* a Brown ortholog is also missing. Interestingly, not a single analyzed insect sequence clusters with the human multidrug efflux transporter ABCG2.

The transporters of the **ABCH** subfamily are missing in humans [6], [8]. To date, the only vertebrate member has been found in zebrafish [113], [114]. All other ABCH proteins have been identified from invertebrate species [29], [52], [115]–[117]. The ABCH subfamily of *C. populi* includes three putative ABC transporters that are highly similar to those of *T. castaneum* (Figure 2).

### RNA-seq Analyses Reveal Tissue-specific Expression of ABC Transporters in Juvenile *C. populi*

**Figure 6:**
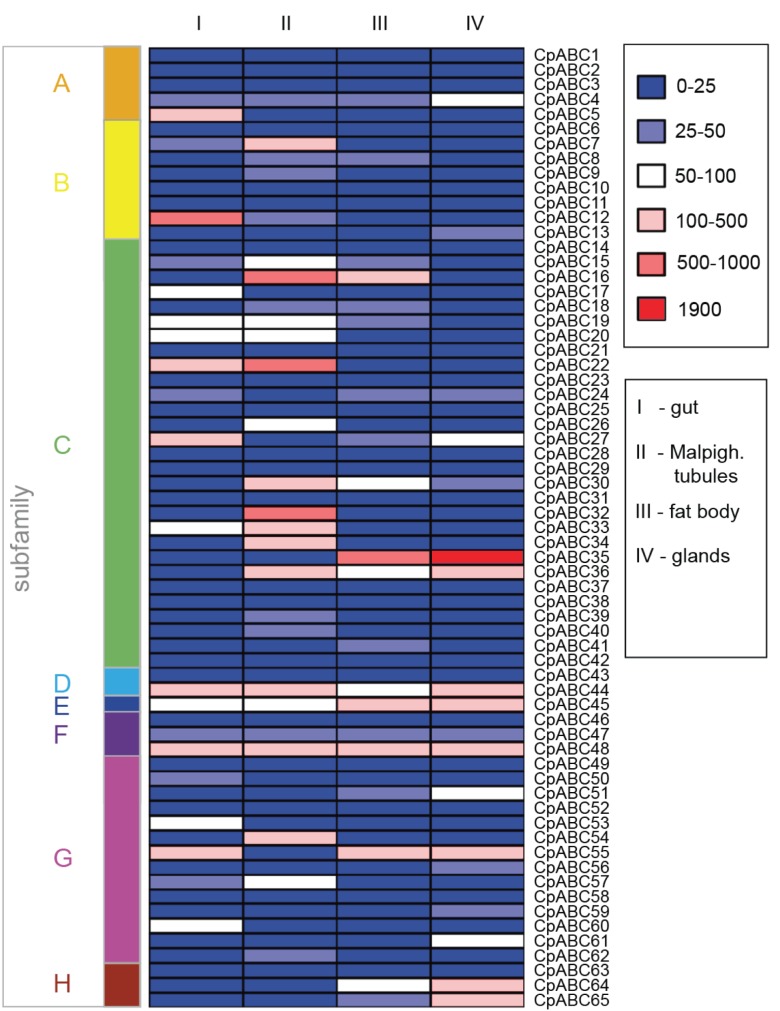
Colormap of the expression profiles of the 65 putative ABC genes of *C. populi*. Values are shown for four different tissues of larvae: gut, Malpighian tubules, fat body and glands. Counts of RNA-seq reads (derived from three replicates for each tissue) normalized to the effective library size and to the length of the corresponding sequences (see Table S2 for data). Expression levels are illustrated by a six grade color scale representing the sequence coverage for each transcript for each tissue, respectively.

To link the above suggested functions for the *C. populi* ABC proteins to those which are differentially expressed in the larval tissues of *C. populi*, we carried out a comprehensive transcriptome sequencing of different tissues dissected from the poplar leaf beetle. All raw sequence data (in the following called reads) are listed in Table S3 and S4. The resulting expression patterns of all identified ABC transporters in intestinal tissue, Malpighian tubules, fat body and defensive glands is depicted in Figure 6. It shows that among the 65 predicted ABC transporters, 43 are expressed at least in one of the tested tissues with more than 25 normalized read counts per base (25-fold sequence coverage). As previously demonstrated in the literature [118], [119], evaluation of the RNA-seq data with quantitative real-time PCR data shows also in our experiments the comparability of the two methods (Figure S3).

Five transcripts were found to be abundant in all tested tissues which suggest their essential role in cellular processes. Among them is, for example, *Cp*ABC4 which was classified as member of the ABCA subfamily. According to our phylogenetic analysis, the closest human homologs, which are involved in lipid translocation, are clustered into group I of the NBD1 branch (Figure 2). Although the NBD2 of *Cp*ABC4 clusters to ABCA12 and 13, the sequence comparison (using BLAST) of the complete sequence supported the homology of *Cp*ABC4 to human ABCA members of group I. Additionally, *Cp*ABC44 an ABCD candidate was highly expressed in all larval tissues, as well. It is homologous to the human ABCD1 and 2 and, therefore, presumably linked to the transport of very long chains of fatty acids in peroxisomes (Figure S2) [102]. Furthermore, we detected in all larval tissues abundantly expressed transcripts encoding soluble ABC proteins: *Cp*ABC45 as a member of the ABCE and *Cp*ABC47 and *Cp*ABC48 members of the ABCF subfamily. Also, in the red flour beetle, the *Tcabce-3a* and *Tcabcf-2a* transcripts were abundant throughout all life stages and highly abundant in the adult intestinal/excretory tissues and carcass [52]. Furthermore, the silencing of *Tcabce-3a* as well as *Tcabcf-2a* resulted in growth arrest and mortality of the beetles. Thus, ABCE and ABCF proteins are essential for cellular functions in all insect tissues including initiation of translation [104], [105] and ribosome biogenesis [120].

In the following, we describe differential expression of putative ABC transporters in the different larval tissues:

#### Gut tissue

We found 17 transcripts abundant in the intestinal tissue of *C. populi* encoding members of the following subfamilies: one sequence of ABCA, two of ABCB, eight of ABCC, five of ABCG (Figure 6). The existence of ABC transporters in the gut influences the absorption and bioavailability of nutrients, water, ions and plant derived compounds. The predicted ABCA subfamily sequence *Cp*ABC5 exhibits a high mRNA level only in the gut tissue. Its deduced protein clusters together with *Tc*ABCA-9A/B of *T. castaneum* (Figure 2). The silencing of these two red flour beetle genes resulted in high mortality and severe defects in wing and elytra development, depending on the developmental stage of treatment. This indicates an essential function for cell physiology, but a ligand has not been identified for these proteins to date [52]. The closest homolog in humans is ABCA3 which is related to phospholipid transfer but also to the modulation of cell susceptibility to chemotherapy of tumors [11], [121], [122]. Thus, *Cp*ABC5 may have a special function in this tissue, in addition to a role in lipid trafficking.

The highest transcript level of ABC transporters in the intestinal tissue was detected for *Cpabc12* which was classified into the subfamily B. It is also expressed in Malpighian tubules but ten times less. *Cp*ABC12 is a full transporter, and possesses most likely homology to human ABCB1 (MDR1, P-glycoprotein), 4, 5 and 11 as well as the *D. melanogaster* Mdr50 (bootstrap value of 63 percent) (Figure 3). Though ABCB4 acts in humans as a transporter for phospholipids in the liver [75], it is involved in the zebrafish’s cellular resistance to noxious chemicals [123]. Except for ABCB11, which is a bile salt transporter [76], all the homologous vertebrate ABCB members can confer multidrug resistance [72]–[74]. We hypothesize a function in the translocation of phytochemicals for *Cp*ABC12 in the gut of *C. populi*. *Cpabc7* is the second ABCB candidate with a high expression level in the gut, albeit not as high as *Cpabc12*. Moreover, *Cpabc7* is 3 times more highly expressed in the Malpighian tubules than in the gut. *Cp*ABC7 is most homologous to the human ABCB6 (Figure 3) which is reported to be localized in the Golgi apparatus [124], mitochondria, plasma membranes [125], vesicular structures [126], or endolysosomes and lysosomes of cells [79]. ABCB6 is discussed to play a role in the heme metabolism [78], [127]–[129], in the drug and arsenite resistance of cells [130]–[132].

All eight ABCC candidates highly expressed in the gut tissue cluster together with human CFTR, SURs, ‘long’ MRPs and ABCC4. This implies a broad substrate spectrum for these insect transporters which, however, cannot be specified further from our phylogenetic analysis (Figure 4, S1).

All five ABCG candidates highly expressed in the larval gut tissue cluster together with the human ABCG1 and ABCG4. These proteins are involved in sterol homeostasis. Among these *C. populi* ABCG sequences, *Cpabc55* showed the most elevated transcript level. Its deduced protein is homologous to *Tc*ABCG-4C (Figure 5) whose involvement in the transport of lipids to the cuticle has been suggested and, thus, that it is required for the formation of a waterproof barrier in the epicuticle [52]. *Cpabc55* is also highly expressed in glands and fat body tissue but not in the Malpighian tubules. The expression of *Tcabcg-4c* was higher in intestinal/excretory tissues than in carcass tissue [52]. The function of the other four ABCG transporters cannot be predicted from our analyses. However, it has been demonstrated recently that an ABCG1-homolog in the fungus *Grossmannis clavigera* confers tolerance to monoterpenes which contributes to the fungus’ ability to cope with the chemical defence of its host plant [133]. Therefore, the ABCG proteins’ specificity in insects may not be limited to sterols or lipids but may have a broader substrate spectrum - that is not known to date. Besides trafficking of physiological substrates, the identified ABC transporters (particularly the ABCC candidates together with the ABCB *Cp*ABC12, and the ABCGs *Cp*ABC50, 53, 60) in the gut tissue may also play a critical role in regulating the absorption of plant secondary metabolites or influence the effectiveness of pesticides in the phytophagous *C. populi*.

#### Malpighian tubules

Insect Malpighian tubules are critical for osmoregulation. Moreover, the tubules have the capability to excrete actively a broad range of organic solutes and xenobiotics, such as insecticides. Recently, we have shown a role of the excretion system in the homeostasis of phytochemicals in the larval body of leaf beetles [39]. Additionally, the tubules play a significant role in immunity by sensing bacterial infections and mounting an effective killing response by secretion of antimicrobial peptides [134]. We found 21 transcripts abundant in the Malpighian tubules of *C. populi* encoding members of the following subfamilies known to contain multidrug resistance proteins: four of ABCB, 14 of ABCC, three of ABCG (Figure 6).

Among the four predicted ABCB members displaying a high mRNA level in the Malpighian tubules, two, *Cp*ABC7 and 12, were already described in the gut section above. The third candidate, *Cp*ABC8, is most similar to human mitochondrial ABCB10 (Figure 3). For ABCB10 different roles have been suggested, including protection against toxic reactive oxygen species, heme synthesis, or peptide transport [77], [135], [136]. For this tissue, we speculate that it is involved in antimicrobial peptide transfer. The forth ABCB protein, *Cp*ABC9, clusters together with the human mitochondrial ABCB7 which is involved in the iron-sulfur cluster assembly essential for multiple metabolic pathways throughout the cell (Figure 3) [80], [81]. RNAi of the homologous *Tc*ABCB-5A demonstrated the pivotal function of this gene in the red flour beetle: its down-regulation resulted in severe morphological defects and high mortality depending on the developmental stage treated [52]. Hence, the three most likely mitochondrial localized ABCB candidates, namely *Cp*ABC7-9, are proposed to be of vital importance in the cells. However, for *Cp*ABC12, which is a full transporter and clusters to human proteins related to xenobiotic resistance, we can predict a similar function in the larval excretion system.

Most putative ABC transporter transcripts identified in *C. populi* are present at a high level in the excretion system of the juvenile beetles compared to the other tissues. Particularly, the 14 candidates belonging to the ABCC subfamily are the most highly transcribed compared to other subfamilies in this tissue. Remarkably, one of the highly expressed candidates, *Cp*ABC16, clusters in our phylogeny together with *Cp*ABC35 (*Cp*MRP) which is involved in the accumulation of plant-derived metabolites (Figure S1) [40]. Therefore, it is tempting to speculate a role for *Cp*ABC16 in the excretion of phytochemicals in *C. populi* larvae.

Among the three candidates of the G-subfamily, two are highly transcribed only in the Malpighian tubules: *Cp*ABC54 is a homolog of *Tc*ABCG-9B from the White group and *Cp*ABC62 is homologous to *Tc*ABCG-9A from the Scarlet group (Figure 5). RNAi targeting *Tcabcg-9a* or *b* resulted in both cases not only in white eyes but also in a whitish appearance of the Malpighian tubules due to the absence of tryptophan metabolites/kynurenine and pteridines. These eye pigment precursors are stored and processed in the larval tubules before being released for further conversion into pigments in the developing adult eyes [137]–[139]. In addition, in *D. melanogaster* White is expressed in intracellular vesicles in tubule principal cells, suggesting that White participates in vesicular transepithelial transport of cGMP [140]. *Cp*ABC57 is the only ABCG candidate that is also expressed in the intestine and belongs to the human ABCG1 and ABCG4 branch (Figure 5). Taken together, the conspicuous overrepresentation of drug-resistance associated proteins, including the ABCC proteins together with the members of the subfamily B (*Cp*ABC12) and G (*Cp*ABC57), in the excretion system suggests a role for these candidates in the extrusion of xenobiotics or phytochemicals from the larval body.

#### Fat body

The fat body of insects is a polymorphic tissue. It performs a vast array of fundamental activities in the intermediary metabolism and is involved in maintaining the homeostasis of hemolymph proteins, lipids, and carbohydrates [141]. Predominantly, the storage of lipid reserves in the form of glycogen and triglycerides is essential in the life of holometabolous insects, primarily in their survival of metamorphosis [142]. In humans, members of the subfamilies A, B, D and G are known to be involved in lipid transport [11], [143]. In principal, we found the expression of ABC transporters in the larval fat body of *C. populi* to be low compared to the other tested tissues (Figure 6). From the ABCB subfamily, we identified in the fat body only *Cpabc8* exhibiting a low transcript level comparable to that of the Malpighian tubules. As described above, it clusters with the human mitochondrial ABCB10 which is associated with different functions, also described above, but not particularly with lipid transfer.

From ABCG we found *Cpabc5*1 and *Cpabc55* with high expression in the fat body. Both deduced proteins cluster to the human ABCG1 and ABCG4 (Figure 5). Only one sequence was exclusively expressed in this body part, namely *Cpabc41*, a member of the subfamily C (Figure 4). Other ABCC members which are highly expressed in this tissue are the homologous *Cpabc16* and *Cpabc35* (Figure S1). *Cp*ABC35 is known to translocate phytochemicals [40].

Noticeably, we found high expression of putative ABCH genes (*Cpabc64, Cpabc65)* in the fat body tissue. Up to now the function of this insect specific subfamily has been unclear. However, RNAi targeting *Tcabch-9c* in the flour beetle revealed a lethal, desiccated phenotype similar to the silencing of *Tcabcg-4c* mentioned above. This ABCH member also seems to be involved directly or indirectly in the transport of lipids from epidermal cells to the cuticle [52]. Based on our data we can hypothesize a role for ABC transporters in phytochemical translocation (by members of the subfamily C and the ABCG candidate), in cuticle formation (by members of the ABCH subfamily) in the fat body, but not particularly in the lipid storage of this tissue. Transporters which are important for this function might be lowly expressed and therefore not detected in our analyses.

#### Defensive glands

The nine pairs of defensive glands enable larvae of *C. populi* to chemically defend themselves *via* deterrent secretions. Each of these dorsal glands is composed of several secretory cells which are attached to a large reservoir. The anti-predatory effect of the secretions can be attributed to salicylaldehyde synthesized within the reservoir by a few enzymatic reactions from the pre-toxin salicin, which is sequestered from the host plant [35], [37]. Recent studies have identified *Cp*ABC35 (*Cp*MRP) which is essential for the sequestration of salicin [40]. It is associated with the accumulation of the plant-derived metabolite in intracellular storage vesicles. Intriguingly, *Cpabc35* is the only predominant transcript in the defensive glands of *C. populi* (Figure 6). Its expression level lies far beyond all other ABC transporters in all tissues. There are four additional predicted ABCC proteins with high expression clustering to the human CFTR, SURs, ‘long’ MRPs and ABCC4, but not particularly to *Cp*ABC35 (Figure 4, S1). In *T. castaneum* another member of this group (not homologues to *Cp*ABC35; Figure 4) has been identified as playing a role in the production of secretions in odiferous stink glands [144]. The silencing of *Tcabcc-6a* (TC015346) in *T. castaneum* resulted in a strong reduction of alkenes in the secretions produced by abdominal and prothoracic glands. Although a substrate for *Tc*ABCC-6A has not been described as yet, the hypothesis can be advanced that ABC transporters functioning in the formation of secretions seem to be a widespread phenomenon in insects.

Besides ABCC proteins, members of the subfamilies B, G and H also have elevated mRNA levels in the defensive glands. *Cp*ABC13 is a member of the B-subfamily exclusively expressed in the defensive glands. It clusters particularly with the human mitochondrial ABCB8 (Figure 3). ABCB8 is known to be responsible for iron transport and doxorubicin resistance in melanoma cells *via* the protection of mitochondrial DNA from doxorubicin-induced DNA damage [145].

Among the five candidates of the ABCG also possessing a high mRNA level in the defensive glands, *Cp*ABC56, 59 and 61 are expressed only in this tissue. *Cp*ABC59 clusters to the human ABCG5:ABCG8 that pump cholesterol and other sterol derivatives, and all of the four other proteins cluster to human ABCG1 and 4, which may have a broader substrate spectrum including xenobiotics (Figure 5).

Remarkably, the expression of putative ABCH genes (*Cpabc64, Cpabc65)* was almost 3 times higher in the glandular tissue compared to the fat body tissue. Owing to this, the two ABCH proteins may have a special function as yet unknown in the defensive glands, but they may also be associated with the formation of the cuticle reservoir for storage of secretions. Furthermore, in the defensive glands there are also ABC candidates potentially associated with the translocation of phytochemicals or other xenobiotics.

### RNAi with Predominant ABC Transporter – *Cpabc35* (*CpMRP*)

Conspicuously, only one ABC sequence, namely *Cpabc35,* displays an exceedingly high transcript level in the defensive glands of *C. populi*. As recently described [40], its function and key role in the sequestration of defensive compound precursors has been demonstrated. In order to test cooperative or compensation effects of other ABC genes, we performed RNAi silencing experiments for *Cpabc35*. Ten days after the injection of *Cpabc35*-dsRNA and *gfp*-dsRNA, glandular tissues were dissected and two biological replicates for each treatment were sequenced. The normalized counts of all transcripts of all samples were calculated. Thereafter, the log_2_ fold-changes of the silenced ABC transporter (*gfp*-injected samples as control) and adjusted p-values were determined using the DESeq package. In all samples (either in RNA-seq or quantitative real-time PCR experiments), we observed varying transcript levels corresponding to the individual biological variance and diversity despite similar developmental stage or living conditions during sample preparation.

The silencing of *Cpabc35* resulted in a significant decrease of its own transcript level (adjusted p-value (padj) = 7.31E-15). One additional ABC transporter, *Cpabc50*, belonging to subfamily G, was determined as differentially expressed (slight upregulation). In non-treated larvae, *Cpabc50* is expressed only in the gut tissue (Figure 6). Its deduced protein clusters together with the human ABCG1 and ABCG4 (Figure 5). However, *Cp*ABC50 could not compensate the function of the salicin translocation into storage vesicles, and, hence, its function remains unclear. Overall, *Cp*ABC35 is an exclusive and highly specific transporter used in the sequestration process, which explains its extraordinarily high transcript level in the defensive glands.

## Conclusion

Phytophagous beetles are adapted to cope with the chemical defense of their host plant. The larvae of the poplar leaf beetle, *C. populi*, evolved the ability to sequester the plant-derived compound salicin and to use it for their own defense against their enemies. The sequestration process proceeds *via* barriers with different selectivity. While the uptake from the gut lumen into the hemolymph together with the excretion by Malpighian tubules is non-selective, the translocation into the defensive glands is selective. In these glands two barriers must be passed: a selective membrane on the hemolymph side and a non-selective membrane on the side towards the cuticle reservoir containing the defensive secretions. Based on our analyses, we predicted specific ABC proteins that are related to the translocation of plant-derived compounds in the larvae. In the gut of *C. populi*, genes of the subfamilies A, B, C and G are predominantly expressed. Almost all of these ABC candidates have been linked in our phylogenetic trees with proteins known to be associated with xenobiotic or drug resistance and which may, therefore, contribute to the non-selective translocation into the larval hemocoel. But depending on the localization of the proteins in the intestinal cells, they may also take part in the detoxification of plant metabolites or pesticides by back-exporting them into the gut lumen. The Malpighian tubules are dominated by candidates of subfamilies B, C and G. In particular, members of the multidrug-related ABCC-group are present in great numbers in this tissue, which suggests a role in the previously observed non-selective phytochemical extrusion by the excretion system. In the defensive glands the salicin-transporting ABCC protein *Cp*ABC35 (*Cp*MRP) is extraordinarily highly expressed in comparison to the other tested tissues. It is localized intracellularly in storage compartments of the gland cells and accumulates salicin in these vesicles for further exocytosis into the glandular reservoir. *Cp*ABC35 has a broad substrate spectrum of phytochemicals and controls the non-selective barrier into the reservoir. The differential expression analysis of *Cp*ABC35-silenced defensive glands in comparison to control samples corroborated the observation that the function cannot be compensated by any other ABC transporter with overlapping substrate selectivity in this particular compartment of the glandular cells. The occurrence of other drug-resistant related ABC transporters in the defensive glands may contribute to the selectivity in the membrane of the hemolymph side of the glandular cells by extruding unused plant-derived compounds from these cells. Thus, ABC transporters are key components in the homeostasis control of phytochemicals in the sequestering poplar leaf beetle larvae.

## Supporting Information

Figure S1 **Phylogenetic tree of subfamilies ABCC (green) and ABCB (magenta).** Some transporters contain two NBDs (NBD1 as *Cp*ABCX_1 and NBD2 as *Cp*ABCX_2), others contain only one NBD. Numbers at nodes represent bootstrap values. *C. populi* (Cp); *H. sapiens* (Hs); *T. castaneum* (Tc), *D. ponderosae* (Dp), *D. melanogaster* (Dm), *B. mori* (Bm); *Microsporida* (Msp).
(EPS)

Figure S2 **Phylogenetic tree of subfamilies D (light blue), E (blue), and F (purple).** Red, *C. populi* (Cp); blue, *H. sapiens* (Hs); black, *T. castaneum* (Tc), *D. melanogaster* (Dm). Star, *T. castanaeum* with phenotype after RNAi. Numbers at nodes represent bootstrap values.
(EPS)

Figure S3 **Relative mRNA levels of selected putative ABC transporters in the different tissues of juvenile** ***C. populi.*** Expression data were determined by carrying out RNA-seq (A) and quantitative real-time PCR (B) experiments (n = 3–4, mean ± SD). *CpEF1alpha* and *CpeIF4a* were used for normalization of transcript quantities. ABC gene subfamilies of *C. populi* are color-coded and grouped by their tissue-specific expression level (orange, ABCA; yellow, ABCB; green, ABCC; purple; ABCF; pink, ABCH).
(EPS)

Table S1 ***De novo*** **assembly of the transcript catalogue of** ***C. populi*** **.** The numbers of assembled transcripts and average length after assembly and reassembly show the usefulness of reassembling.
(XLSX)

Table S2 **Accession numbers of homologous sequences added to** ***C. populi*** **'s ABC transporter sequences to calculate phylogenetic trees.**
(XLSX)

Table S3 **Overview of the raw sequence data.** The table exhibits the RNA derived specimens, number of reads, sequencing technology and sequencing mode.
(XLSX)

Table S4 **List of 65 predicted ABC transporters of** ***C. populi*** **.** Accession numbers and sequence length of cDNAs encoding putative ABC transporters of *C. populi* and their corresponding read counts normalized to the effective library size as well as to the sequence length of all ABC transporters in the different larval tissues are presented.
(XLSX)

Table S5 **Primer sets used in quantitative real-time PCR and RNAi experiments.**
(XLSX)

Table S6 **Predicted domain distribution in the deduced protein sequences of all identified ABC transporters of** ***C. populi*** **.**
(XLSX)
